# Commentary: Is So-Called “Split Alpha” in EEG Spectral Analysis a Result of Methodological and Interpretation Errors?

**DOI:** 10.3389/fnins.2021.726912

**Published:** 2021-09-24

**Authors:** Elzbieta Olejarczyk, Aleksander Sobieszek

**Affiliations:** Nalecz Institute of Biocybernetics and Biomedical Engineering, Polish Academy of Sciences, Warsaw, Poland

**Keywords:** split alpha peak, EEG, spectral analysis, Fourier transform, instantaneous frequency, Empirical Wavelet Transform, Hilbert Transform, Matching Pursuit method

## Introduction

The phenomenon of “split alpha” discussed in this paper refers to the existence of two or more frequency peaks in close frequencies, appearing in the frequency power spectrum of EEG record within the range of alpha frequency waves (8–13 Hz) (Chiang et al., 2008, 2011; Olejarczyk et al., 2017). The presence of “split” was predicted in a model of alpha rhythm generation (Robinson et al., 2001, 2003; O'Connor and Robinson, 2004; Xiong and Yao, 2005; Gray and Robinson, 2013). This is the phenomenon appearing with variable intensity, especially in conditions of disturbances of the activity of neuronal networks. In our previous study we discussed the methodological requirements important for more effective detection of “split alpha” and evaluation of the localization of its generators, such as the significance of the EEG window size and localization of the reference electrode during EEG recording (Olejarczyk et al., 2017). Our findings have been questioned in the study published by Zalewska (2020). The author stated that the split alpha effect is nothing but a methodological error caused by spectral leakage. The aim of this commentary is the presentation of the results of FFT analysis supporting the validity of the conclusions formulated in our original paper (Olejarczyk et al., 2017).

## Significance of the Choice of an Appropriate Window Size in the FFT Analysis of Non-Stationary Signals

The spectral analysis based on the Fast Fourier Transform (FFT) is commonly used for a long time in the EEG signal analysis (Grass and Gibbs, 1938; Gibbs and Grass, 1947). Due to the instability of the EEG signals leading to power spectrum changing over time, the Short-Time Fast Fourier Transform (STFFT) was introduced (Grochenig, 2001). However, the application of the STFFT has some limitations related to the use of a finite-time window. Depending on the specific application, windows of various shapes such as Hann, Hamming, Bartlett, Blackman, or Kaiser and other windows are used (Harris, 1978). They differ in the main lobe width, the roll-off rate, and peak side lobe level. The use of finite-time windows causes the effect of spectral leakage, resulting in spreading of undesired frequencies to the whole spectrum. The minimum and maximum resolvable frequencies that can be analyzed using a finite-time window are determined by the Rayleigh and the Nyquist frequencies, respectively. The Rayleigh frequency is given by the inverse of the EEG window size, while the Nyquist frequency is equal to half of the sampling frequency. One of the disadvantages of the FFT transform is a fixed resolution, i.e., a wide window gives better frequency resolution but poor time resolution and vice-versa (Matteo and Talavera, 2018).

## Comparison of the FFT With Other Methods of Non-Stationary Signals Analysis

The simplest method of frequency estimation for narrow-band signals is the zero crossing method (Giannakopoulos and Pikrakis, 2014; Xue et al., 2017). In this paper, we applied a variant of the zero-crossing method suitable for nonstationary signals. It consists of finding successive zero-crossings of the band-pass filtered signal. The distance between two consecutive zero-crossing times is considered a half period of the sinusoidal-like signal, easily translated into frequency.

The next method was based on the application of the Empirical Wavelet Transform and the Hilbert Transform (Huang et al., 1998; Gilles, 2013; Bhattacharyya et al., 2018). First, the Empirical Wavelet Transform method was performed to decompose the analyzed signal into amplitude and frequency modulated components, so-called intrinsic mode functions. Then, the Hilbert transform of each component was applied to estimate the instantaneous amplitude and the instantaneous frequency. The functions *EWT1D* and *EWT_InstantaneousComponents*, implemented in the EWT software, were used to perform these calculations (https://www.mathworks.com/matlabcentral/fileexchange/42141-empirical-wavelet-transforms). The standard parameters were set.

Moreover, we applied the Matching Pursuit method consisted in the construction of the EEG signal from three Gabor functions to obtain the time-frequency energy distribution of the analyzed EEG segment (Blinowska and Durka, 2001). The calculations were performed using Svarog software (https://git.braintech.pl/brain/svarog2/).

## Discussion: Significance of the Spectral Analysis in Evaluation of the EEG Patterns

**Figure 1:**
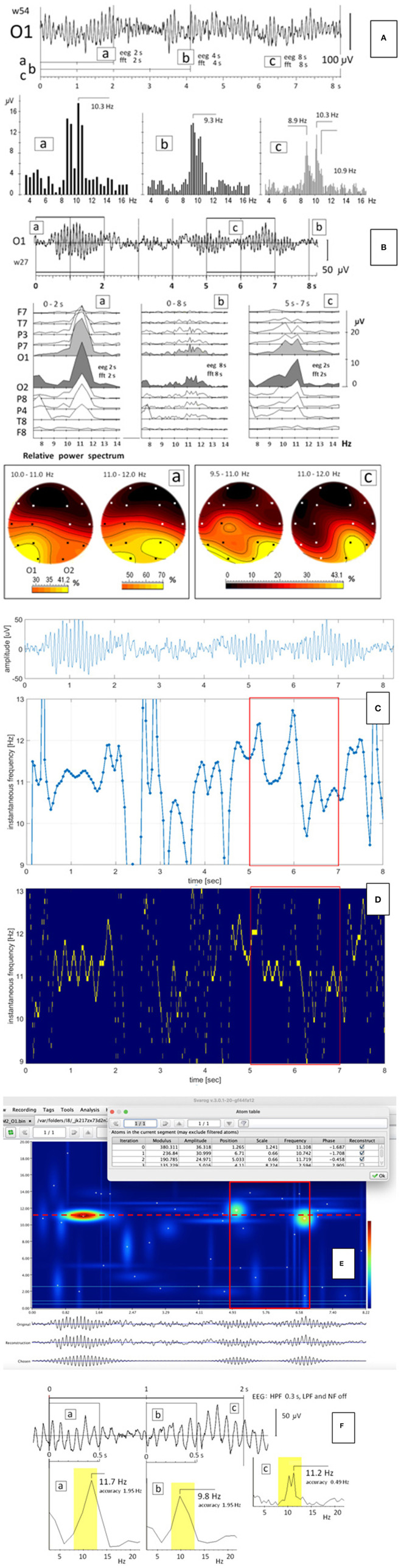
**(A)** EEG segment with relatively stable pattern of the alpha split; a, b, c – the FFT calculated for 2-, 4-, and 8-s windowed segments of the EEG record starting at time zero; **(B)** EEG segment (from Figure 1 presented in our original paper) recorded in another person; a, c – the FFT (upper panel) and the relative power spectrum (lower panel) calculated for two 2-s windows in time ranges from 0 to 2 s and from 5 to 7 s, respectively. The FFT and the EEG windows had the same size; b – the FFT calculated for the 8-s EEG segment; **(C–F)** the instantaneous frequency **(C)**, the Empirical Wavelet Transform with the Hilbert Transform **(D)**, and the Matching Pursuit method **(E)** and the FFT **(F)** calculated for the same EEG segment as in **(B)**. The 2-s window in which the split alpha was visible in **(B-**c), was marked as the red rectangle.

The original study was performed in a group of 27 ambulatory patients (23 females, four males; mean age: 29.9 ± 11.5 years) with headache, fainting, loss of consciousness or epilepsy. Split alpha is a normal phenomenon that occurs frequently in the EEG signals of all ambulatory patients. The split alpha was observed in 271 out of 1,620 consecutive 2-s EEG segments moved by 200 ms (17% of segments of the whole record). However, the basic information necessary for solving the discussed problem, i.e., to determine if this effect is an artifact due to the spectral leakage or a physiological nature phenomenon, may be received from the results of analysis of the two short fragments of EEG records illustrated in Figure 1. The original EEG records were analyzed during preparation of the previous paper (Olejarczyk et al., 2017). The EEG was recorded using the DigiTrack Elmiko System with standard parameters of analog filters: 0.3–70 Hz and notch filter with sampling frequency of 250 Hz. The FFT analysis was performed using the software implemented in the Elmiko System.

The EEG signals may be very non-stationary. In our previous study, we illustrated on the spatiotemporal map that the process of split-alpha formation may take less than a second (Olejarczyk et al., 2017). Sometimes, this phenomenon is more stable. An example of such conditions is shown in Figure 1A. The EEG segment shown on Figure 1A is more stable than the EEG segment shown on Figure 1B, which is reflected in the FFT of these signals. The FFT of the EEG signal shown on Figure 1A was performed for windows of 2, 4, and 8 s, all starting at time zero (Figures 1A-a,b,c). A clear split-alpha with maxima at 8.9 and 10.3 Hz is visible independently on window size. The frequency resolution depends on the FFT window size: for the FFT window of 2 s is equal to 0.5 Hz (Figures 1A-a); for 4 s, 0.25 Hz (Figures 1A-b); and for 8 s, 0.125 Hz (Figures 1A-c). Since the frequency difference between two maxima in the split alpha was 1.4 Hz, in each of these cases, the frequency resolution was sufficient to say that this is not a spectrum leakage but the split alpha effect. These results are in accordance with the conclusions formulated by Zalewska (2020).

However, the formation of the split alpha is a dynamic process that requires usually the evaluation of the EEG signal in a wider spatial and temporal context. The split alpha may appear and disappear continuously and what is important, it may appear simultaneously on different electrodes, which is clearly seen in the fragment of the EEG signal illustrated on Figure 1B.

The FFT of the second EEG segment was performed for three windows (marked with appropriate frames in Figure 1B): two 2-s EEG windows in time range from 0 to 2 s (Figures 1B-a) and from 5 to 7 s (Figures 1B-c), and the 8-s EEG window in time ranges from 0 to 8 s (Figures 1B-b). Both EEG and FFT windows had the same size. The split alpha is clearly visible only in the FFT of the 2-s window in Figures 1B-c, but its presence is evident neither in the 2-s window in Figures 1B-a nor the 8-s window, which contains both 2-s windows (Figures 1B-b). The split alpha is observed also in the larger window but its amplitude is much smaller than that in the 2-s window. The EEG signal is not stationary. Figure 1F explains clearly that the split alpha is generated as a result of a disintegration of the rhythm alpha. At the beginning, the frequency of the alpha rhythm was equal to 11. 7 Hz (Figures 1F-a). Then, the rhythm slowed down to 9.8 Hz (Figures 1F-b). Of course, in the FFT of the whole 2-s segment, both components must appear, which is visible as the split alpha (Figures 1F-c). The alpha rhythm disintegration is clearly visible also in the time-frequency distribution obtained using the Matching Pursuit method (Figure 1E).

The existence of the split-alpha effect is supported by its presence in several EEG derivations at the same time with amplitudes depending on the localization of the split alpha generators (Figures 1B-c). In this EEG segment a hemispheric asymmetry is clearly visible. The split-alpha is expressed better in the left than in the right hemisphere with the maximal amplitude in the occipital lobe, mainly at higher frequencies in the right hemisphere.

The maps of the relative power spectrum (Figures 1B-a,c) illustrate well the formation of the split alpha as a result of the interaction between two generators placed in the posterior region of the brain. At the beginning, the maximal relative power spectrum was observed in the left occipital lobe (O1) in the frequency range of 10–11 Hz, and in the left occipital lobe (O1) and in the right parieto-occipital lobe (P8, O2) in the frequency range of 11–12 Hz (yellow spots in Figures 1B-a). Later, the generator localized in the right hemisphere dominated mainly in the range of 11–12 Hz, which resulted in the split-alpha generation (yellow spots in Figures 1B-c).

Additionally, the calculation of the instantaneous frequency using three other methods: the zero-crossing method, the Empirical Wavelet Transform with the Hilbert Transform, and the Matching Pursuit method, was performed for the EEG signal presented in Figure 1B. The results of all these methods confirmed the results obtained using the FFT. In Figures 1C–E, two clearly different frequency levels can be distinguished in the same 2-s window in which the split alpha was visible in Figures 1B-c,F.

The study of the split alpha effect has some limitations. Further studies should be performed for various physiological and pathological states of the brain to evaluate the impact of brain condition on the split alpha effect. Moreover, it should be verified if this effect is specific for the alpha band range or it can appear in other frequency bands also. We analyzed clinical EEG data that were recorded with a standard sampling frequency of 250 Hz, but future studies could be performed with higher sampling frequency of EEG signals as well. Finally, it would be interesting to perform the analysis of intracranial EEG to investigate this effect. However, the main purpose of this commentary was to explain better the methodological issues related to the FFT application discussed in the publication of Zalewska (2020).

## Conclusions

Presented results of FFT analysis of the selected EEG fragments clearly illustrate that the “split alpha” is not a result of methodological and interpretation error. The optimal window size enabling detection of such transient events like “split alpha,” with good frequency resolution (0.5 Hz), was 2 s. The detection of split alpha in longer windows is possible only in case of enough long temporary stability of the EEG patterns such as those shown in Figure 1A. Thus, in the case of the non-stationary signals, especially containing the components of very close frequency, other methods like the zero-crossing method, the Matching Pursuit method, and the Empirical Wavelet Transform with the Hilbert Transform may be more reliable. The results of the FFT analysis presented in our studies are consistent with the results of visual evaluation of the patterns of EEG records performed in a wide temporal and spatial context.

## Author Contributions

EO: conception of the work, EEG analysis, making Figures 1C–E, and writing of the manuscript. AS: conception of the work, interpretation of EEG data, making Figures 1A,B,F and critical revision of the manuscript. All authors contributed to the article and approved the submitted version.

## Conflict of Interest

The authors declare that the research was conducted in the absence of any commercial or financial relationships that could be construed as a potential conflict of interest.

## Publisher's Note

All claims expressed in this article are solely those of the authors and do not necessarily represent those of their affiliated organizations, or those of the publisher, the editors and the reviewers. Any product that may be evaluated in this article, or claim that may be made by its manufacturer, is not guaranteed or endorsed by the publisher.
